# A Strategy for Simultaneous Engineering of Interspecies Cross-Reactivity, Thermostability, and Expression of a Bispecific 5T4 x CD3 DART^®^ Molecule for Treatment of Solid Tumors

**DOI:** 10.3390/antib14010007

**Published:** 2025-01-17

**Authors:** Renhua R. Huang, Michael Spliedt, Tom Kaufman, Sergey Gorlatov, Bhaswati Barat, Kalpana Shah, Jeffrey Gill, Kurt Stahl, Jennifer DiChiara, Qian Wang, Jonathan C. Li, Ralph Alderson, Paul A. Moore, Jennifer G. Brown, James Tamura, Xiaoyu Zhang, Ezio Bonvini, Gundo Diedrich

**Affiliations:** MacroGenics Inc., Rockville, MD 20850, USA; spliedtm@macrogenics.com (M.S.); kaufmant@macrogenics.com (T.K.); gorlatovs@macrogenics.com (S.G.); baratb@macrogenics.com (B.B.); shahk@macrogenics.com (K.S.); gillj@macrogenics.com (J.G.); kurt.stahl@zymeworks.com (K.S.); jennifer.dichiara@astrazeneca.com (J.D.); qwang09@amgen.com (Q.W.); jonathanchli@hotmail.com (J.C.L.); rlphldrsn@yahoo.com (R.A.); paul.moore@zymeworks.com (P.A.M.); brownj@macrogenics.com (J.G.B.); tamuraj@macrogenics.com (J.T.); zhangx@macrogenics.com (X.Z.); bonvinie@macrogenics.com (E.B.)

**Keywords:** bispecific antibodies, DART, phage display, 5T4, thermostability, expression, T-cell engager, target-mediated drug disposition

## Abstract

**Background:** Bispecific antibodies represent a promising class of biologics for cancer treatment. However, their dual specificity and complex structure pose challenges in the engineering process, often resulting in molecules with good functional but poor physicochemical properties. **Method:** To overcome limitations in the properties of an anti-5T4 x anti-CD3 (α5T4 x αCD3) DART molecule, a phage-display method was developed, which succeeded in simultaneously engineering cross-reactivity to the cynomolgus 5T4 ortholog, improving thermostability and the elevating expression level. **Results:** This approach generated multiple DART molecules that exhibited significant improvements in all three properties. The lead DART molecule demonstrated potent in vitro and in vivo anti-tumor activity. Although its clearance in human FcRn-transgenic mice was comparable to that of the parental molecule, faster clearance was observed in cynomolgus monkeys. The lead α5T4 x αCD3 DART molecule displayed no evidence of off-target binding or polyspecificity, suggesting that the increased affinity for the target may account for its accelerated clearance in cynomolgus monkeys. **Conclusions:** This may reflect target-mediated drug disposition (TMDD), a potential limitation of targeting 5T4, despite its limited expression in healthy tissues.

## 1. Introduction

Bispecific antibodies (BsAbs) represent a promising class of biologics that can revolutionize the treatment of different diseases by simultaneously targeting two antigens. Compared to monoclonal antibodies (mAbs), BsAbs offer several advantages, including increased potency, reduced toxicity [1], faster internalization [2], and the ability to treat diseases with new mechanisms of action [3]. These benefits, however, come with additional challenges. Unlike mAbs, BsAbs often require modifications such as linkers, additional domains, or mutations to ensure the proper integration and pairing of the two binding specificities [3,4]. These modifications can impact various properties of the bispecific molecules, such as thermostability (referred to as stability here on), expression [5], pharmacokinetics (PK) [6], and immunogenicity [7]. The interplay between the two specificities within BsAbs is also complex, further complicating the development of these molecules [5]. Considering that properties such as affinity, stability, and polyspecificity are interconnected and can influence each other, engineering BsAbs can be a tedious and time-consuming endeavor. To streamline the process, it is essential to adopt an engineering approach that addresses multiple properties simultaneously. For example, computational modeling was employed to engineer both the stability and affinity of mAbs concurrently [8]. Other approaches relied on the inclusion of a heating step [9], a thermo-stable cell line (CHO) [10], or the use of liability-free CDR sequences [11] to engineer multiple properties simultaneously.

The largest group of BsAbs are T-cell engagers (TCEs) [12]. Most T-cell engaging BsAbs work by co-engaging CD3-expressing T-cells and tumor antigen-expressing cancer cells. This interaction triggers a T-cell-mediated killing of the cancer cells, bypassing the conventional T-cell activation process. Multiple TCEs have been approved for targeting liquid tumors [13], and two TCEs have been approved for targeting solid tumors [14,15].

**Figure 1:**
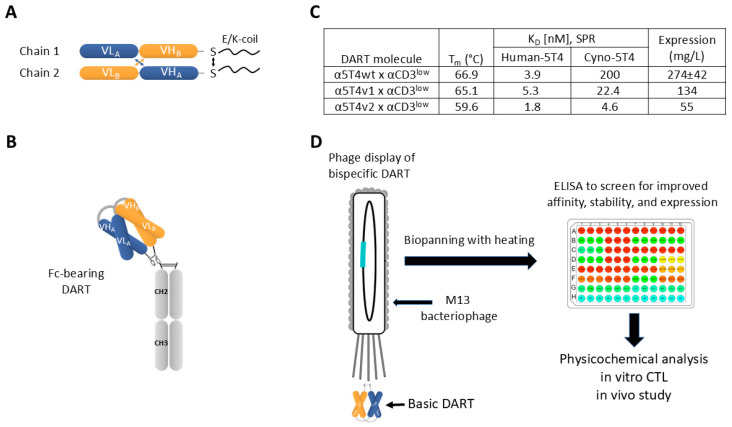
DART molecule structure and workflow to improve biophysical properties of 5T4 x CD3 DART molecules. (**A**). Schematic showing the design and components of the basic DART molecule. The light chain of antibody A (VL_A_) is linked to the heavy chain of antibody B (VH_B_) and expressed as one chain, which pairs with a second chain consisting of the light chain of antibody B (VL_B_) and the heavy chain of antibody A (VH_A_). C-terminal of the VH domain in each chain is a cysteine residue and a charged coil (either K- or E-coil), which stabilize the structure. (**B**). The structure of an Fc-bearing DART molecule, which consists of the basic DART unit and the CH2 and CH3 of an Fc domain. (**C**). Physicochemical properties of α5T4wt x αCD3^low^ and two variants. Melting temperature T_m_, affinities to human and cyno 5T4 as determined by surface plasmon resonance (SPR), and the titer in supernatants of transiently transfected CHO cells after elution from protein-A resin are shown. (**D**). Workflow of the experimental design. The basic DART molecules displayed on M13 phage were screened to identify variants with improved properties. Selected variants were further characterized by various bioanalytical, in vitro, and in vivo methods.

We have utilized the DART platform [16] to develop TCEs for liquid [16,17] and solid tumor indications [18]. A DART molecule is a diabody stabilized by an engineered disulfide bond. It relies on a short linker and oppositely charged coiled-coil sequences (E/K coils) [19] to form a tightly packed structure (Figure 1A). The short distance (approximately 30 Å) between the two antigen binding sites and the rigidity of the DART structure ensure an efficient crosslinking of target and effector cells by TCEs of the DART format [20,21]. The objective of our study was to develop a DART molecule that redirects T-cell cytotoxic activity towards tumor cells expressing the 5T4 tumor antigen while also possessing physicochemical properties and a pharmacokinetic (PK) profile suitable for clinical development. The overexpression of 5T4 leads to tumor progression and drug resistance [22] and is associated with poor patient prognosis in ovarian, gastric, lung, and colorectal cancers [22,23]. Our initial lead molecule had a significantly higher affinity for human 5T4 than the cynomolgus monkey ortholog (cyno 5T4), making it challenging to predict human PK and preclinical safety. Previous attempts to enhance the affinity of the initial lead to cyno 5T4 involved the panning of a phage-displayed Fab library of the 5T4 antibody. Upon the reformatting of the selected Fab variants with improved affinity into the DART format, the stability and expression of the DART molecules were compromised. To improve the interspecies cross-reactivity while maintaining stability and expression, in this study, we decided to engineer the 5T4 antibody in DART format and create two phage-displayed DART libraries. By introducing a heating step during the biopanning process, screening for high display levels, and strong binding to cyno 5T4, we selected variants with enhanced affinity, improved stability, and expression. From the pool of newly generated variants, we identified a lead DART molecule that mediated robust anti-tumor activity against multiple 5T4-expressing tumor cell lines, as confirmed by in vitro cytotoxic T lymphocyte (CTL) assays and in vivo xenograft mouse models. In human FcRn-transgenic mice, the lead DART molecule retained a similar PK profile as the original parent molecule. However, when administered to cynomolgus monkeys, this DART molecule displayed faster clearance than the parental molecule with lower affinity towards cyno 5T4. Since no off-target binding or polyspecificity was observed, we surmised that the improvement in cyno-5T4 affinity resulted in target-mediated drug deposition (TMDD), exposing a potential general challenge for the development of 5T4-targeted therapeutics.

## 2. Materials and Methods

### 2.1. Plasmids, Recombinant Proteins, and Cell Lines

Anti-CD3 antibody hXR32 was derived from clone SP34 by humanization [16]. Anti-5T4 antibody 5E6 was developed at MacroGenics by standard hybridoma technology and humanized by CDR grafting to yield h5E6.

For phage display, the two genes of the α5T4 x αCD3^low^ [24] basic DART molecules were cloned into a bicistronic phagemid vector, similar to pMORPH18 [25]. Transcription of the two DART chains is driven by a single lacZ promoter. The open reading frames had the following structure: OmpA signal sequence-αCD3-VL-linker-α5T4-VH-linker-K coil; PhoA signal sequence-α5T4-VL-linker-αCD3-VH-linker-E coil-linker-g3 protein of phage M13. The linker between the E-coil and the g3 coat protein contained a trypsin cleavage site. M13 K07 helper phage with a trypsin cleavage site in the g3 protein [26] was used.

Plasmid pCIneo (Promega, Madison, WI, USA, cat #: E1841) and ExpiCHO cells (Thermo Fisher, Waltham, MA, USA, cat #: A29133) were used for mammalian expression of DART molecules [19] that were fused to a FC domain. The extracellular domains of human and cynomolgus monkey 5T4 were cloned into UCOE plasmid (EMD Millipore, Burlington, MA, USA, cat #: UC0E02) with a C-terminal His-tag and expressed in stable ExpiCHO cells and purified by Ni-NTA chromatography. Cell lines A498 (cat #: HTB-44), HCT-116 (cat #: CCL-247), H1650 (cat #: CRL-5883), and MDA-MB-231 (cat #: HTB-26) were obtained from ATCC (Manassas, VA, USA) and maintained in ATCC-recommended growth medium. The identity of the cell lines was confirmed by Short Tandem Repeat (STR) analysis [27].

### 2.2. Construction of Phage Libraries to Engineer 5T4 Antibody h5E6

Library 1 with targeted mutations in VH-CDR2 and VH-CDR3 of h5E6 was generated by Kunkel mutagenesis [28]. Details of the mutagenesis, transformation of electrocompetent cells, and rescue of the phage library were described previously [29]. In brief, stop codons were introduced into position 58 of CDR2 and position 100 of CDR3 of the VH gene. The mutated phagemid was converted into single-stranded DNA by transformation of E. coli strain CJ236 (Thermo Fisher, cat #: NC0492648) and infection with M13-K07 helper phage, which was generated at MacroGenics and encodes the full-length minor coat protein-III. The single-stranded DNA and two degenerate oligonucleotides were used to generate double-stranded recombinant DNA. The degenerate oligonucleotides introduced mutations in h5E6 at positions 57–61 in VH-CDR2 and positions 100–102 in VH-CDR3. These positions encoded sequences enriched in the biopanning experiment in which α5T4v1 and α5T4v2 were isolated.

Library 2 was generated by error-prone PCR, as described previously [30], on both the VH and VL of α5T4v2. Briefly, the mutazyme (Agilent, Santa Clara, CA, USA, cat #: 200550) was used to amplify the VH and VL in a mutagenic buffer of the following composition: 7 mM MgC12, 50 mM KC1, 10 mM Tris (pH 8.4), 0.01% gelatin (wt/vol), 0.2 mM dGTP, 0.2 mM dATP, 1 mM dCTP, 1 mM TTP, and 0.1 mM MnCl2. The cycling parameter was one cycle at 95 °C for 2 min, followed by 34 cycles at 95 °C for 30 s, 60 °C for 15 s, 72 °C for 15 s, and a final extension step of 72 °C for 2 min. The resultant VH and VL DNA fragments were stitched together by overlapping PCR [31], with the linking DNA fragment that includes VH and VL sequences of αCD3^low^. The product of overlap PCR was digested and subcloned into the phagemid vector to create the final DNA library.

The DNA libraries produced by Kunkel mutagenesis (library 1) and error-prone PCR (library 2) were both subjected to electroporation into TG1 electrocompetent cells (LGC Biosearch Technologies, Hoddesdon, UK, cat #: 60502) to create the bacterial libraries, which were grown and infected with M13-K07 helper phage to create the phage libraries as described [29].

### 2.3. Biopanning of the Phage Library

Three rounds of biopanning were performed against cyno 5T4 as described previously [29], with decreasing concentrations of biotinylated cyno 5T4 in each round: 10 nM, 2 nM, and 1 nM, respectively. In the first round, no heating step was included. In the second round of biopanning, the harvested phage particles were heated at 50 °C (for library 2) or 52 °C (for library 1) for 1 h and then mixed with cyno 5T4 at a concentration of 2 nM for biopanning. In the third round, the phage particles were heated at 54 °C (for library 2) or 58 °C (for library 1), and biopanning was conducted with 1 nM of cyno 5T4. After the second and third round of biopanning, the output was spread as bacterial single colonies on 2xYT agarose plates with 2% glucose and 50 µg/mL carbenicillin. After overnight incubation at 37 °C, single colonies were picked and grown in a 96-deep well plate to produce phage particles for phage ELISA.

### 2.4. Phage ELISA to Select for Variants with Improved Affinity, Stability, and Expression

Phage supernatant in 96-deep well plates was clarified by centrifugation. To subject the phage to heat challenge, 100 µL of phage supernatant from each well was transferred to a PCR plate and heated at 58 °C (library 1) or 54 °C (library 2) for 1 h. Ten microliters of heat-treated phage particles and untreated phage particles were diluted in 90 µL of PBS casein (Thermo Fisher, cat #: 37528) and applied to Nunc Maxisorp plates (Thermo Fisher, cat #: 437111), coated with biotinylated cyno 5T4 at 1 µg/mL or an anti-EK-coil antibody (generated at MacroGenics) at 2 µg/mL. The binding signal of the anti-EK-coil antibody was used to quantitate the level of DART expression on phage surface. Binding was detected with an anti-M13 antibody conjugated to horse radish peroxidase (HRP) (1:5000 dilution; Sino Biological, Beijing, China, cat #: 11973-MM05T-H), followed by addition of a chemiluminescent substrate (SuperSignal™ ELISA Pico Substrate, Thermo Fisher, cat #: 37070). The binding values of heated vs. unheated samples were used to select variants for improved stability. The binding values to cyno 5T4 were divided by those to the anti-EK-coil antibody to screen for variants with improved binding to cyno 5T4. The binding values to the anti-EK-coil antibody were used to select variants with good expression levels.

### 2.5. Expression in ExpiCHO Cells and Purification of DART Molecules

Selected variants were cloned into pCIneo plasmid to be expressed as Fc-bearing DART molecules. The expression of selected variants was evaluated in a 96-deep well plate. A total of 500 ng plasmid DNA in 10 µL distilled H2O was mixed with 0.5 µL FectoPRO of Polyplus (Sartorius AG, Göttingen, Germany, cat #: 101000019) and 0.5 mL ExpiCHO cells. The plate was then incubated for 5–6 days at 900 RPM and 37 °C with 8% ambient CO_2_. After the incubation period, the culture supernatant was harvested by centrifugation and subsequently cleared by filtering through 96-well plate filter (Pall corporation, New York, NY, USA, cat #: 8165). The expression was quantified by ELISA with the cleared culture supernatant, which was diluted 50- to 200-fold and applied to a microtiter plate coated with goat anti-human-Fc polyclonal antibodies (ImmunoReagents Inc., Raleigh, NC, USA, cat #: GtxHu-004-D) and detected by HRP-conjugated goat anti-human-Fc polyclonal antibody (ImmunoReagents Inc., cat #: GtxHu-004-DHRPX). Expression at larger scale in shake flasks and purification of DART molecules were carried out as described previously [19].

### 2.6. Physicochemical Characterization of DART Molecules

The purified proteins of selected variants were analyzed on a Biacore-T100 instrument (Cytiva, Marlborough, MA, USA) to measure the affinities. A F(ab’)_2_ fragment of goat anti-human IgG Fc antibody (Jackson Immunoresearch, West Grove, PA, USA, cat #: 109-006-008) was immobilized on a CM5 sensor chip (Cytiva, cat #: 29149603) for DART capture. DART molecules were injected at a flow rate of 100 µL/min to achieve a captured protein level of approximately 400 resonance units (RU). In the association phase, human or cyno 5T4 protein was injected in duplicate at concentrations of 0, 62.5, 250, and 1000 nM for 120 s, followed by the dissociation phase. Binding was analyzed in buffer containing 10 mM HEPES (pH 7.4), 150 mM NaCl, and 0.005% P20 surfactant. Reference curves were obtained by injecting each dilution of 5T4 protein over the treated surface without prior immobilization of the DART molecules. Binding curves at zero concentration were subtracted as a blank. The dissociation equilibrium constant (K_D_) was calculated by globally fitting the association/dissociation curves using the 1:1 Langmuir interaction model (BIAevaluation software version 4.1, Cytiva).

Melting temperatures (T_m_) were measured using differential scanning calorimetry (DSC) on a VP Capillary DSC instrument (Malvern Panalytical, Worcestershire, UK). The protein concentration employed for all measurements was 1 mg/mL in PBS buffer. Thermal scans were performed from 15 °C to 95 °C at a heating rate of 1 °C/min. Thermograms were obtained after subtracting the buffer scan and fitting to a non-2-state model using Origin 7.0 software (OriginLab, Northampton, MA, USA).

The percentage of monomeric DART molecules after elution from the MabSelect SuRe column (Cytiva, cat #: 17543802) was analyzed by SEC-HPLC (ACQUITY UPLC BEH 200Å; Waters Corp, Milford, MA, USA) at a flow rate of 0.4 mL/min.

### 2.7. In Vitro Evaluation of the Anti-Tumor Activity

PBMCs were separated from heparinized whole blood collected from healthy donors (StemExpress, Rockville, MD, USA) by gradient centrifugation using Ficoll-Paque Plus (Cytiva, cat #: 17144003). For CTL assays, PBMCs (1 × 10^5^/well) were cocultured with target cells at 10:1 ratio in the presence of 10-fold serial dilution of test article in culture medium in a 96-well plate. After 48 h, culture supernatants were collected. Target cell lysis was measured using the CytoTox 96 non-radioactive cytotoxicity assay kit (Promega, cat #: G1780) following the manufacturer’s protocol. Cytokine concentrations were measured using standard ELISA kits (R&D systems, Minneapolis, MN, USA) according to the manufacturer’s protocol.

### 2.8. Evaluation of Anti-Tumor Activity in Cell-Derived Xenograft Mouse Models

All studies were reviewed and approved by MacroGenics’ Institutional Animal Care and Use Committee (IACUC). Six- to eight-week-old NSG MHCI-/- mice (strain 23848) were obtained from Jackson Laboratory (Bar Harbor, ME, USA) and maintained at MacroGenics’ animal facility. Mice were immune-reconstituted by retro-orbital injection of 8 × 10^6^ human PBMCs. Seven days later, 5 × 10^6^ HCT-116 or MDA-MB-231 tumor cells were implanted subcutaneously in a 1:1 mixture with Matrigel. When tumors grew to 100 mm^3^, test articles were administered once a week intravenously via tail vein injection. The tumor volumes were recorded for the period of 32 days, and the growth curves were plotted with the average tumor volume of 8 mice in each group. The body weight of mice was also recorded throughout the experiment as a measure of drug toxicity.

### 2.9. PK Study in Human-FcRn Transgenic Mice and Cynomolgus Monkeys

DART molecules were injected intravenously at 5 mg/kg into groups of five human FcRn-transgenic mice (Tg32 scid strain 018441, Jackson Laboratory, RRID: IMSR_JAX: 018441). Blood samples were collected before injection and 0.08, 6, 24, 48, 72, 168, 216, 312, 360, and 576 h after injection. The collected blood samples were mixed with Rexxip A buffer (Thermo Fisher, cat #: NC9920015) at a 1:10 ratio for storage before analysis by ELISA.

Studies in cynomolgus monkeys were conducted at contract testing facilities, Altasciences Preclinical Seattle (Everett, WA, USA) and Inotiv (Mt. Vernon, IN, USA). Studies were designed in accordance with the US Department of Agriculture Animal Welfare Act (9 CFR Parts 1, 2, and 3) and the Guide for the Care and Use of Laboratory Animals (Institute of Laboratory Animal Resources) and were approved by the Institutional Animal Care and Use Committee of the testing facility. The DART molecules were administered to the animals via intravenous infusion for a duration of 30 min (α5T4v3 x αCD3^low^) or 2 h (α5T4wt x αCD3^low)^ at a dose of 1 or 10 mg/kg. Two variants of α5T4v3 x αCD3^low^, with or without YTE mutations [32] in the Fc region, were administered. Peripheral blood samples were collected prior to dosing and at periodic time points out to approximately 168 h after dosing. Blood samples were processed to obtain serum and were frozen.

In the ELISA, the blood samples were further diluted in storage buffer and applied to microtiter plates coated with human 5T4-His protein or polyclonal goat anti-idiotype antibodies against CD3-antibody (generated at MacroGenics). Binding was detected with an HRP-labeled anti-human Fc secondary antibody (Jackson Immunoresearch, cat #: 109-035-008).

### 2.10. Evaluation of Off-Target Binding by Membrane Proteome Array

Off-target binding of α5T4v3 x αCD3^low^ was evaluated using the Membrane Proteome Array (MPA) platform (Integral Molecular, Philadelphia, PA, USA) [33]. The MPA comprises ~6000 different human membrane proteins, including human 5T4 and CD3-epsilon, that were individually transfected and overexpressed in QT6 cells. Thirty-six hours post-transfection, transfected cells were stained with α5T4v3 x αCD3^low^ (1.25 μg/mL), and DART binding was detected with a fluorescently labeled secondary antibody.

### 2.11. Evaluation of Polyspecificity by Insulin and DNA ELISA

Two variants of α5T4v3 x αCD3, with or without YTE mutations in the Fc region, were evaluated in insulin and DNA ELISAs as described previously [34]. In brief, 96-well plates of Nunc Maxisorp (Thermo Fisher, cat #: 442404) were coated overnight at 4 °C with insulin (10 μg/mL) (Sigma-Aldrich, St. Louis, MO, cat #: 19278) or DNA (100 ng/mL) (Sigma-Aldrich, cat #: D1626) in phosphate-buffered saline (PBS) pH 7.2 (Thermo Fisher, cat #: 20012027). Wells were washed three times with water, blocked with PBS containing 1% bovine serum albumin (Rockland Immunochemicals, Pottstown, PA, USA, cat #: BSA-10) for 1 h at room temperature. After washing with water, 50 μL of test article (10 μg/mL) was added in duplicate to the wells and incubated for 1 h at room temperature. Plates were washed three times with water, and goat anti-human IgG-Fc conjugated to HRP (Jackson ImmunoResearch, cat #: 109-035-008) was added at 1:5000 dilution to each well. Plates were incubated for 1 h at room temperature, washed with water, and signal was developed by adding 80 μL of TMB substrate (SURMODICS, Eden Prairie, MN, USA, cat #: TMBC-1000-01) to each well. Reactions were stopped after approximately 4 min by adding sulfuric acid to each well, and absorbance was read at 450 nm with Victor Luminometer (Perkin Elmer, Waltham, MA, USA). Insulin and DNA-binding scores were calculated as the ratio of the ELISA signal of the antibody at 10 μg/mL to the signal of a well containing PBS buffer only.

## 3. Results

5T4 is an attractive tumor antigen for targeted cancer therapy due to its overexpression in several solid tumors and limited expression in normal tissues [35]. To enable the therapeutic targeting of 5T4 with TCEs, we combined the V domains of humanized monoclonal antibodies to 5T4 (h5E6) and CD3 (hXR32), respectively, to generate a bispecific α5T4 x αCD3 DART molecule (Figure 1A,B). The affinity of the parental h5E6 antibody (α5T4wt) to cyno 5T4 is approximately 50-fold lower than to human 5T4 (Figure 1C), thus limiting the use of cynomolgus monkeys to evaluate its safety and PK. Previous attempts to affinity-optimize the parent antibody, which was engineered in Fab format by mutagenesis with NNS codons, resulted in two variants, α5T4v1 and α5T4v2, with improved binding to cyno 5T4 (Figure 1C). Reformatting these α5T4 variants into Fc-bearing α5T4 x αCD3 DART molecules maintained the improved binding to cyno 5T4. However, the new variants destabilized the DART structure, resulting in lower expression and reduced stability compared to the DART molecule with the parental α5T4wt (Figure 1C).

### 3.1. Engineering Interspecies Cross-Reactivity, Stability, and Expression of α5T4 in DART Format

We decided to further engineer α5T4 in DART format in conjunction with the partnering CD3 specificity. This involved presenting the α5T4 x αCD3 as a basic DART molecule on the surface of M13 phage (Figure 1D). Two phage libraries of such DART molecules were subjected to biopanning, and phage enzyme-linked immunoassay (ELISA) was conducted to screen for variants with improved cross-reactivity to cyno 5T4, stability, and expression.

M13 phage is resistant to temperatures up to 80 °C [36], which is higher than the melting temperature (T_m_) of DART molecules. DART molecules with increased stability can, therefore, be isolated from M13-displayed DART libraries by incubating the library at high temperatures.

**Figure 2:**
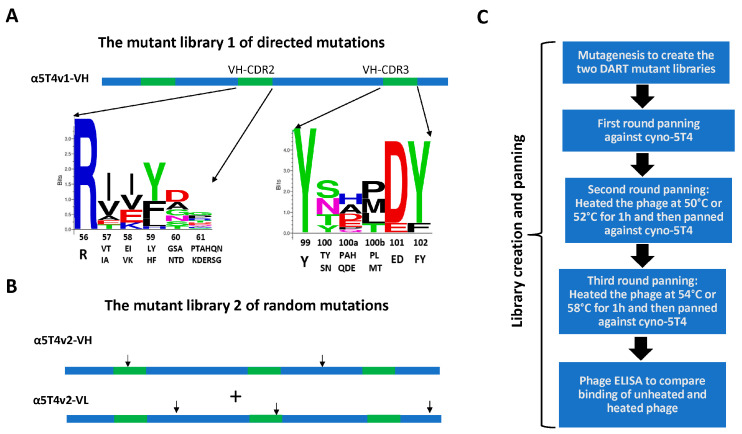
Design and biopanning of the two mutagenic phage DART libraries. (**A**). The design and sequence logo of a site-directed mutagenic library (library 1). Kunkel mutagenesis was performed to randomize positions 57–61 in VH-CDR2 and positions 100–102 in VH-CDR3. Sequence logos of the randomized regions in library 1 prior to biopanning are shown. Kabat positions and amino acid residues used for diversifying these positions are listed below them. (**B**). The α5T4v2 variant (VH and VL) was used as a template for error-prone PCR to create a mutant library with random point mutations (library 2), which are indicated by the arrows. (**C**). Workflow of the experimental steps from DART library creation, biopanning to ELISA screening.

To identify the desired α5T4 variants, two mutagenic libraries were generated (Figure 2A,B). In library 1, the positions 57–61 in VH-CDR-2 and 100–102 of VH-CDR-3 of α5T4v1 were randomized simultaneously with two degenerate oligonucleotides. Both oligonucleotides were designed to encode amino acid residues in the randomized positions that were enriched in the biopanning experiment in which α5T4v1 and α5T4v2 were isolated (Figure 2A). The resulting library had a size of 2.3 × 10^8^, providing greater than 10-fold coverage of all potential 1.8 × 10^7^ combinations. In library 2, random point mutations were created in the frameworks and CDRs of the VH and VL genes of α5T4v2 by error-prone PCR with an error-prone DNA polymerase (mutazyme) in a mutagenic buffer. This PCR created a library of 1 × 10^8^ variants that carry an average of 2.3 mutations per VH or VL gene (6.8 mutations per kilobase). Before screening the libraries, the biopanning conditions were optimized. Phage particles displaying α5T4 x αCD3 DART molecules with α5T4wt or α5T4v1 variants were heated at different temperatures, and the integrity of the antigen binding site was analyzed by phage ELISA with 5T4-coated plates (Figure S1). Both DART molecule variants remained stable at 50 °C but gradually unfolded at higher temperatures. The DART molecule with α5T4v1 had lower 5T4 reactivity at 58 °C and 60 °C than the DART molecule with α5T4wt (Figure S1B), consistent with the lower T_m_ of the α5T4v1 x αCD3^low^ (65.1 °C), compared to that of α5T4wt x αCD3^low^ (70 °C). 

**Figure 3:**
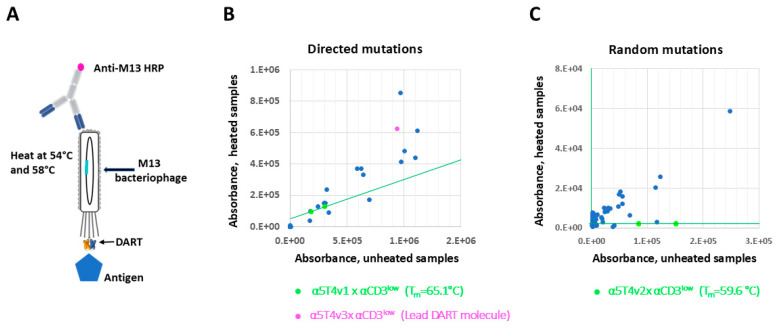
Phage ELISA to screen for variants with improved stability. (**A**). A schematic of the ELISA with heated phage to screen for stable variants. The antigen coated on the microtiter plate is used to capture the phage particles that are either heated or unheated, followed by detection with anti-M13 antibody conjugated to horse radish peroxidase (HRP). (**B**). Dot plot of phage ELISA with selected variants from library 1 (directed mutations) plus parent molecule (α5T4v1 x αCD3^low^). Phage supernatants of variants were used directly or were heated at 58 °C for 1 h prior to ELISA. Phage supernatants of α5T4v1 x αCD3^low^ were assayed in duplicate to obtain a trendline (green line). The variants above the trendline retained binding better and thus were more stable than the parent molecule. The variant in magenta color is the lead DART molecule, α5T4v3 x αCD3^low^. (**C**). Equivalent dot plot as in B. for variants selected from library 2 (random mutations). Variants above the green line retained binding better than the parent molecule α5T4v2 x αCD3^low^ after being heated at 54 °C for 1 h.

Three rounds of biopanning were conducted with both libraries, using decreasing concentrations of cyno 5T4 (10 nM, 2 nM, and 1 nM in rounds 1, 2, and 3, respectively; Figure 2C). The second and third rounds of biopanning included a heating step. Due to the difference in the T_m_ of the starting DART molecules from the two libraries, the heating temperatures for the libraries were adjusted to 52 °C and 58 °C for library 1 and 50 °C and 54 °C for library 2, respectively. Ninety-two variants from the second or third round of biopanning were randomly selected from each library and subjected to phage ELISA to assess their binding to cyno 5T4 and to an anti-EK-coil antibody (Figure 3 and Figure S2). Since the EK coil is conserved in all DART variants, binding to the anti-EK-coil antibody was used to normalize the DART concentration of the different phage variants. To select variants with enhanced stability, phage particles were also heated at 54 °C or 58 °C, respectively, and then analyzed for binding to cyno 5T4 by ELISA.

To select variants with improved stability, expression, and affinity, we employed the following criteria. First, the selected variants needed to retain binding better than the parent molecule when heated (variants above the green lines in Figure 3B,C). The library based on α5T4v2 yielded a higher percentage of such improved variants, potentially due to its lower starting T_m_ compared to α5T4v1 (59.6 °C vs. 65.1 °C). Next, variants need to maintain good expression on the phage, as indicated by the high ratio values of heated sample/unheated in the dot plot of Figure 3 and the high binding signal to the anti-EK-coil antibody in the phage ELISA (bottom plate of Figure S2A). Based on our previous findings from other research projects, we observed that the expression of DART molecules on the phage correlated well with their expression in CHO cells, as exemplified by a high-expression control phage in Figure S2A. Lastly, variants with high ratios of cyno-5T4/anti-EK-coil binding signals (Figure S2B) were favored. Applying these selection criteria, eleven unique variants, all obtained from library 1, were chosen for further characterization.

### 3.2. Physicochemical Characterization of Isolated Variants

**Figure 4:**
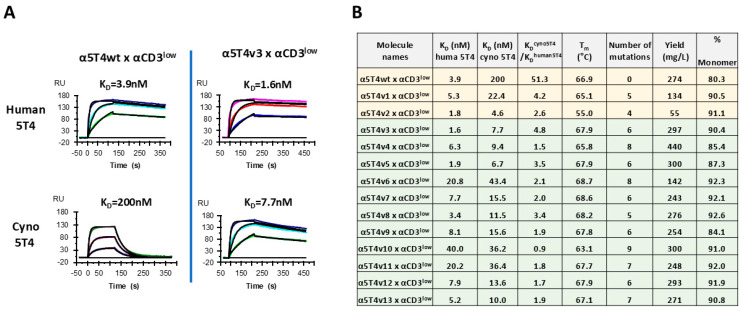
Physicochemical properties of selected variants. (**A**). Affinity measurement by SPR of α5T4wt x αCD3^low^ and α5T4v3 x αCD3^low^ to human or cyno 5T4. (**B**). Physicochemical properties of selected variants: affinities to human or cyno 5T4, cyno/human affinity ratio, melting temperature T_m_, number of the mutations in comparison to α5T4wt x αCD3^low^, titer in supernatants of transiently transfected CHO cells, and % monomer after elution from protein-A resin. The three parent DART molecules are listed in yellow rows; the new variants are listed in green rows.

All selected basic DART variants were converted to Fc-bearing DART molecules and expressed in CHO cells. The DART molecules were then purified for physicochemical characterization, including the measurement of affinity by surface plasmon resonance (SPR) (Figure 4A and Figure S3) and the determination of T_m_ by differential scanning calorimetry (DSC) (Figure 4B and Figure S4). As indicated in Figure 4B, most of the selected variants demonstrated similar cross-reactivity to cyno 5T4 when compared to the two parental DART molecules with α5T4v1 or α5T4v2 and exhibited significant improvement over the DART molecule with the original α5T4wt. Notably, all but two of the variants displayed higher T_m_ than all three parent molecules (highlighted in yellow). Additionally, except for the DART molecule with α5T4v6, the expression levels of the variants nearly doubled in comparison to the DART molecules with α5T4v1 or α5T4v2. The monomer profiles (% monomer after protein-A column) remained similar before and after engineering and did not correlate with either T_m_ or expression level.

A variant with improved stability and similar affinity as α5T4v2 was identified from the library of random mutations (library 2). This variant had a point mutation (F36L, Figure S5B) in the framework-2 region of the light chain, and a computational model revealed this mutation was buried within the DART molecule (Figure S5C). When this VL variant was combined with seven VH variants, the expression level of five DART molecules (v1, v2, v5, v7, and v8) was further improved in both 0.5 mL and 560 mL cultures (Figure S5A).

Based on the overall balanced physicochemical properties and the lack of sequence liabilities, α5T4v3 x αCD3^low^, α5T4v5 x αCD3^low^ (with F36L), and α5T4v8 x αCD3^low^ were selected as lead DART molecules for further characterization.

### 3.3. In Vitro Cytotoxic Activity Against a Panel of Cancer Cell Lines

**Figure 5:**
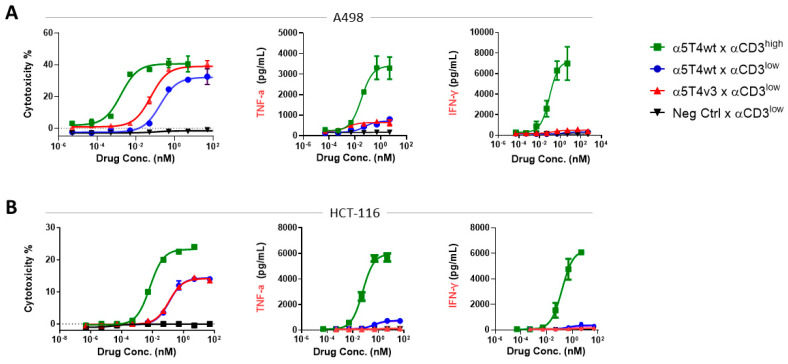
In vitro T-cell-mediated tumor cytotoxicity and cytokine secretion of 5T4 x CD3 DART molecules. Lysis of A498 kidney cancer cells (**A**) or HCT-116 (**B**) and associated secretion of TNF-α and IFN-γ to evaluate the potency of α5T4v3 x αCD3^low^ in comparison to α5T4wt x αCD3^high^ and α5T4wt x αCD3^low^. PBMCs were mixed with the tumor cells at a ratio of 10:1 with various concentrations of the DART molecules. After 48 h, the number of live tumor cells was analyzed by flow cytometry, and the cytokine production was measured by ELISA.

The biological activity of three selected lead molecules was first evaluated in CTL assays using multiple tumor cell lines as target cells. Among the three selected DART molecules, α5T4v3 x αCD3^low^ efficiently induced the lysis of various cancer cell lines, including A498 (renal cancer) and HCT-116 (colon cancer) (Figure 5A,B), and other cell lines (Table S1). The half maximal effective concentration (EC_50_) of target cell lysis ranged from double- to single-digit picomolar. We compared the activity of α5T4v3 x CD3^low^ to two benchmark constructs, α5T4wt x αCD3^high^ and α5T4wt x αCD3^low^. Both benchmarks contained the same parental 5T4 antibody but differed in the affinity of their CD3 specificity: 8 nM for CD3^high^ [16] versus 205 nM for CD3^low^ [24]. α5T4wt x αCD3^low^ and α5T4v3 x αCD3^low^ exhibited similar CTL activities against multiple cell lines, indicating that the improved cross-reactivity to cyno 5T4 did not compromise anti-tumor potency towards tumor cell lines expressing human 5T4.

Compared to α5T4wt x αCD3^high^, both DART molecules with the αCD3^low^ showed lower potency (higher EC_50_ values) in CTL assays, as anticipated. Their maximal cytotoxic activity (E_max_), however, was not compromised, while inducing minimal cytokine secretion of tumor neurosis factor-alpha (TNF-α), interferon-gamma (IFN-γ, Figure 5), interleukin-2 (IL-2), and interleukin-6 (IL-6) (Figure S6), potentially resulting in better tolerability in vivo than a DART molecule with αCD3^high^ [37].

To assess whether the improved cyno cross-reactivity of α5T4v3 translated to similar reactivity on cells expressing human or cyno 5T4, we compared the potency of 5T4 × CD3 DART molecules containing either α5T4wt or α5T4v3 in triggering CD3 signaling. This was evaluated using a T-cell activation reporter assay with target cells expressing human or cyno 5T4. In such assays, α5T4v3 x αCD3^low^ exhibited similar EC_50_ values against both cell lines compared to α5T4wt x αCD3^low^, which had a 6-fold difference (Figure S7).

### 3.4. In Vivo Anti-Tumor Activity in Cell-Line-Derived Xenograft Models in Mice

**Figure 6:**
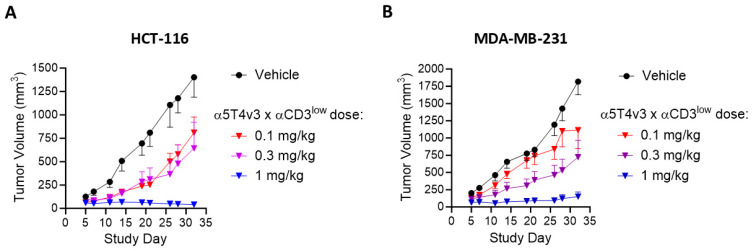
Anti-tumor activity of α5T4v3 x αCD3^low^ in cell-line-derived xenograft models. (**A**). In vivo studies of α5T4v3 x αCD3^low^ in HCT-116 xenograft model. Human PBMCs were injected into NSG/MHCI-/- mice at day 0, followed by injection of tumor cells at day 7. α5T4v3 x αCD3^low^ was administered once a week starting at day 7. (**B**). In vivo studies of α5T4v3 x αCD3^low^ in MDA-MB-231 xenograft model.

The anti-tumor activity of the lead molecule, α5T4v3 x αCD3^low^, was evaluated in humanized mouse models. NOD/SCID/IL2-γ chain KO (NSG) mice were engrafted with human tumor cell lines and reconstituted with human peripheral blood mononuclear cells (PBMCs), followed by treatment with α5T4v3 x αCD3^low^ after tumors had been established. The tumor xenograft models were generated with HCT-116 cells and MDA-MB-231 cells, which express low and medium levels of human 5T4, respectively (Table S1). The α5T4v3 x αCD3^low^ DART molecule showed dose-dependent anti-tumor activity in both xenograft models (Figure 6), with a dose of 1 mg/kg completely inhibiting tumor growth and a dose of 0.3 mg/kg mediating partial activity. In xenograft models of HCT-116 cells, both α5T4wt x αCD3^low^ and α5T4v3 x αCD3^low^ exhibited comparable anti-tumor activity (Figure S8) at 0.1 mg/kg, consistent with the similar potencies in CTL assays and similar affinities towards 5T4 by SPR.

### 3.5. PK Studies in Human-FcRn Transgenic Mice and Cynomolgus Monkeys

The PK of α5T4v3 x αCD3^low^ and α5T4wt x αCD3^low^ was assessed in human-FcRn transgenic mice and cynomolgus monkeys. The 5T4 specificity in these DART molecules is not cross-reactive to its target in mice. Clearance in mice is, therefore, solely determined by the molecule’s physicochemical properties and serum stability, as well as any potential polyspecificity. By contrast, both 5T4 and CD3^low^ specificities of these molecules are cross-reactive to their targets in monkeys; therefore, the PK profiles of these DART molecules in monkeys may be influenced by target recognition.

**Figure 7:**
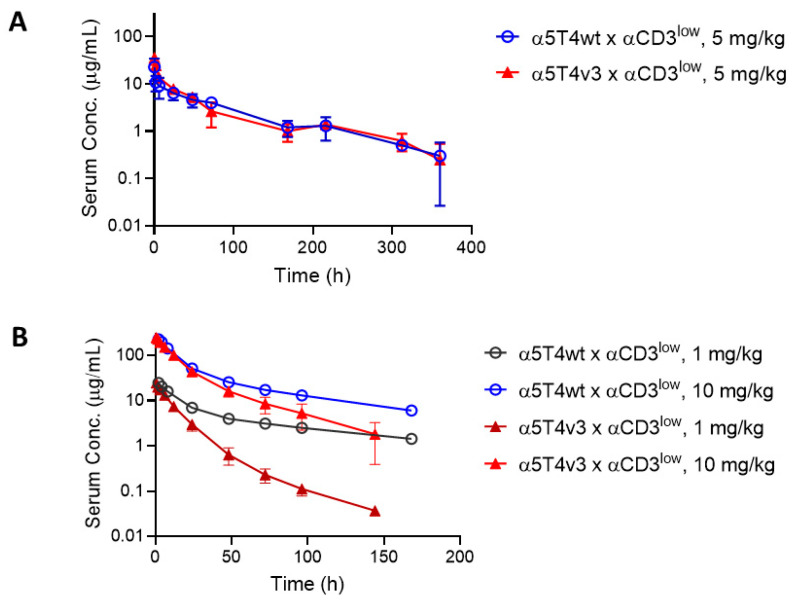
Pharmacokinetic (PK) studies of α5T4wt x αCD3^low^ and α5T4v3 x αCD3^low^ in human FcRn-transgenic mice and cynomolgus monkeys. (**A**). PK studies comparing the clearance of α5T4wt x αCD3^low^ and α5T4v3 x αCD3^low^ in human FcRn transgenic mice. DART molecules were administered intravenously at 5 mg/kg, and blood samples were collected at various timepoints after injection. The plasma concentrations of the DART molecules at each time point were measured by ELISA, and the values shown are the mean concentrations of five mice in each study. (**B**). PK studies conducted in cynomolgus monkeys to compare the clearance rates of α5T4wt x αCD3^low^ and α5T4v3 x αCD3^low^. Two animals were used for each molecule.

As depicted in Figure 7A, both molecules exhibited similar PK profiles in mice, suggesting that the engineering step did not affect the serum stability or polyspecificity of the molecule. In contrast, in cynomolgus monkeys, α5T4v3 x αCD3^low^ exhibited lower serum concentrations than α5T4wt x αCD3^low^ when administered at 1 mg/kg (Figure 7B). A higher dose of 10 mg/kg, however, improved the PK of α5T4v3 x αCD3^low^ to a level comparable to that of α5T4wt x αCD3^low^ (Figure 7B), suggesting a saturable phenomenon. In addition, the variant of α5T4v3 x αCD3^low^ with YTE mutations [32] in its Fc domain failed to improve the PK in the monkeys. Taken together, these findings suggest that the enhanced interaction of α5T4v3 x αCD3^low^ with cyno 5T4 is associated with reduced serum drug levels in cynomolgus monkeys, consistent with target-mediated drug disposition (TMDD).

**Figure 8:**
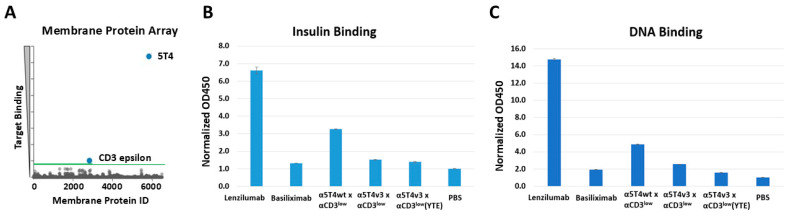
Evaluating the off-target binding and polyspecificity of 5T4 x CD3 DART variants. (**A**). Binding of α5T4v3 x αCD3^low^ to an array of 6000 membrane proteins expressed in QT6 cells was evaluated by flow cytometry. Membrane proteins with a specific binding signal are highlighted in blue (5T4 and CD3 epsilon). Specific binding is defined as a signal > 3 standard deviations above the mean background value (indicated by the green line). (**B**). Insulin binding ELISA. The histogram represents the fold change of binding to insulin over the baseline binding without the test article. For α5T4v3 x αCD3^low^, variants with and without YTE mutations in the Fc domain were included in the assays. Lenzilumab and basiliximab are control antibodies with high or low non-specific binding, respectively. The error bar is the standard deviation of two replicate measurements. (**C**). DNA binding ELISA. Data were processed and summarized as for the insulin ELISA.

Alternative reasons that might lead to accelerated clearance were also investigated. The potential introduction of the antigen spreading of α5T4v3 x αCD3^low^ was ascertained against a membrane protein array consisting of approximately 6000 members. The screening results revealed strong binding to human 5T4 and low binding to CD3, consistent with its measured affinity for either target. At the same time, no binding to any of the other 6000 membrane proteins was detected (Figure 8A). Therefore, off-target binding is unlikely to be the cause of its rapid clearance. The potential polyspecificity of the DART molecule was assessed by measuring its binding to charged molecules [34]. The α5T4v3 x αCD3^low^ showed slightly lower binding to insulin and DNA than α5T4wt x αCD3^low^ (Figure 8B,C). Its binding signal was similar to that of basiliximab (anti-CD25) and much lower than that of lenzilumab (anti-GM-CSF)—two antibodies with low and high polyspecificity [34,38], respectively. The lack of off-target binding and polyspecificity further supports the conclusion that the enhanced affinity of α5T4v3 x αCD3^low^ to cyno 5T4 is responsible for its accelerated clearance in monkeys via TMDD.

## 4. Discussion and Conclusions

Bispecific antibodies have gained popularity as therapeutic reagents in various clinical applications. To achieve the desired mechanism of action, an affinity modulation of the therapeutic antibody is often necessary. For example, the activity of blocking antibodies can be enhanced by increasing the affinity to their targets, while agonistic antibodies often acquire better signaling activity with faster off-rates [39]. While engineering affinity itself is a well-established process, other properties such as stability, expression, or polyspecificity may be adversely affected during affinity optimization [40,41]. Some studies even suggest that there are obligatory trade-offs between affinity and stability when replacing certain amino acids [42,43]. Optimizing the affinity and the CMC characteristics, such as expression and stability, is an essential part of therapeutic antibody discovery and engineering as it will reduce the complexity of development and manufacturing and lower the overall cost of antibody therapeutics.

Our previous attempt to optimize the affinity of the tumor-targeting arm of a α5T4 x αCD3 DART molecule involved the biopanning of a phage-displayed 5T4 antibody library in Fab format. The selected 5T4 Fab variants maintained improved 5T4 binding upon reformatting into DART molecules, albeit with reduced stability and unacceptably low expression. The implementation of several modifications of the selection and screening approach enabled us to identify DART variants with improved affinity and CMC characteristics: (i) the library of 5T4 variants was designed in basic DART instead of Fab format, thus avoiding the potential loss of CMC attributes when converting selected variants from the screening to the final format; (ii) the phage library was incubated at high temperature before biopanning, thus favoring the selection of thermostable DART molecules; (iii) the selected phage particles were screened for 5T4 binding as well as display intensity levels, favoring the selection of DART molecules with improved expression.

Several DART variants with increased affinity for cyno 5T4 and improved stability and expression were isolated from the library with CDR-targeted mutations (library 1), from which α5T4v3 x αCD3^low^ was chosen as the lead molecule. By contrast, DART molecule variants isolated from the library of random mutations (library 2) exhibited improved stability but without an increase in affinity for cyno 5T4. Most of the improved variants from library 2 had mutations spanning both VH and VL, covering both framework and CDR regions.

Many TCEs have shown promising anti-tumor activity, but their clinical development has often been hampered by severe adverse events, especially cytokine release syndrome [44]. Most TCEs that have entered clinical trials bind CD3 on T-cells with high affinity. However, triggering the cytolytic activity of T-cells has a lower activation threshold than triggering the secretion of cytokines. It is, therefore, possible to detune the affinity of the CD3-engaging arm and design TCEs that preferentially induce cytolytic activity without excessive cytokine secretion [45]. The α5T4 x αCD3 DART molecules described here support this concept. α5T4wt x αCD3^high^, with high CD3 affinity, exhibited higher in vitro and in vivo anti-tumor potency than α5T4v3 x αCD3^low^. However, at high drug concentrations, both DART molecules achieved comparable maximum anti-tumor activities. α5T4wt x αCD3^high^ also triggered higher levels of cytokine secretion. In contrast, cytokine secretion induced by α5T4v3 x αCD3^low^ was comparably low, even at the high drug concentrations required for maximal cytolysis. These data suggest that modulating the CD3 affinity is an effective approach to reduce toxicity and improve the therapeutic index of TCE therapeutics.

Although we were able to improve the developability and maintain the potency of the bispecific antibody in this study, the lead molecule, α5T4v3 x αCD3^low^, had rapid clearance and a non-linear PK profile in cynomolgus monkeys. Its serum concentration dropped much faster than that of the parental molecule (α5T4wt x αCD3^low^), which has a 26-fold lower affinity to cyno 5T4 (200 nM for α5T4wt vs. 7.7 nM for α5T4v3). The poor PK profile of the lead molecule was unlikely to have been caused by polyspecificity or off-target binding, as demonstrated by the lack of binding to charged molecules (insulin, DNA) or to other membrane proteins in a 6000-member array. Moreover, the clearance of α5T4v3 x αCD3^low^ in the mouse, a targetless species for this molecule, was much slower than in cynomolgus monkeys. Based on these observations, we hypothesize that the rapid clearance of this DART molecule in monkeys is affinity-dependent and driven by TMDD.

Consistent with other publications [23,35], we could not detect a significant expression of 5T4 in normal adult human and monkey tissues by immunohistochemistry, suggesting that 5T4 might be present in some normal tissues or cell types with a large mass but at a very low expression level, below detectability by immunohistochemistry. Consistent with our findings, non-linear PK was reported with other 5T4-targeting antibodies in humans and non-human primates [46,47]. One notable exception is a recently published study of a tetravalent bispecific 4-1BB x 5T4 agonist with a serum half-life of 5.1 to 9.5 days in cynomolgus monkeys [48]. This bispecific molecule had a relatively low affinity of 66 nM to 5T4. This report suggests that the TMDD of 5T4-targeting antibodies may be minimized by designing molecules with dual 5T4 binding arms of modest affinity to limit or avoid binding to normal tissues with low density of 5T4 receptors while still maintaining avidity-driven [49,50] binding to tumor cells with a high density of 5T4 receptors. An alternative approach to overcome TMDD could be masking the 5T4 binding arm with a peptide cleavable by tumor protease, minimizing binding to normal tissues [51]. Mask cleavage by proteases can restore the binding to the target in the tumor environment.

Taken together, these findings suggest that antibody-based therapeutics targeting 5T4 are likely to face the challenges of TMDD-driven PK and undesirable off-tumor binding. Such challenges can be potentially addressed by protein engineering. The ability to simultaneously screen for multiple properties described here may facilitate the discovery of molecules with the desired features.

## Data Availability

The data presented in the study are included in the article and Supplementary Materials.

## Supplementary Materials

The following supporting information can be downloaded at: https://www.mdpi.com/article/10.3390/antib14010007/s1.
