# Supplementary material for: A Strategy for Simultaneous Engineering of Interspecies Cross-Reactivity, Thermostability, and Expression of a Bispecific 5T4 x CD3 DART® Molecule for Treatment of Solid Tumors
Source: Antibodies (Basel). 2025 Jan 17;14(1):7. doi: 10.3390/antib14010007 (PMC11755548; doi:10.3390/antib14010007)
Supplement: Supplementary file 1 [file antibodies-14-00007-s001.zip › antibodies-3292232-Supplementary Materials.pdf]

Supplementary Materials

A Strategy for Simultaneous Engineering of Interspecies Cross-Reactivity, Thermostability, and Expression of a Bispecific 5T4 x CD3 DART® Molecule for Treatment of Solid Tumors

Renhua R. Huang * †, Michael Spliedt, Tom Kaufman, Sergey Gorlatov, Bhaswati Barat, Kalpana Shah, Jeffrey Gill, Kurt Stahl ‡, Jennifer DiChiara §, Qian Wang ‖, Jonathan C. Li, Ralph Alderson, Paul A. Moore ¶, Jennifer G. Brown, James Tamura, Xiaoyu Zhang, Ezio Bonvini and Gundo Diedrich *

* Correspondence: huangr@macrogenics.com (R.R.H.); diedrichg@macrogenics.com (G.D.)
† Lead contact.
‡ Current affiliation: Zymeworks, Bellevue, WA 98004, USA.
§ Current affiliation: AstraZeneca, Gaithersburg, MD 20878, USA.
‖ Current affiliation: Amgen, Rockville, MD 20850, USA.
¶ Current affiliation: Zymeworks, Vancouver, BC V5T 1G4, Canada.

Supplemental Figures and Legends

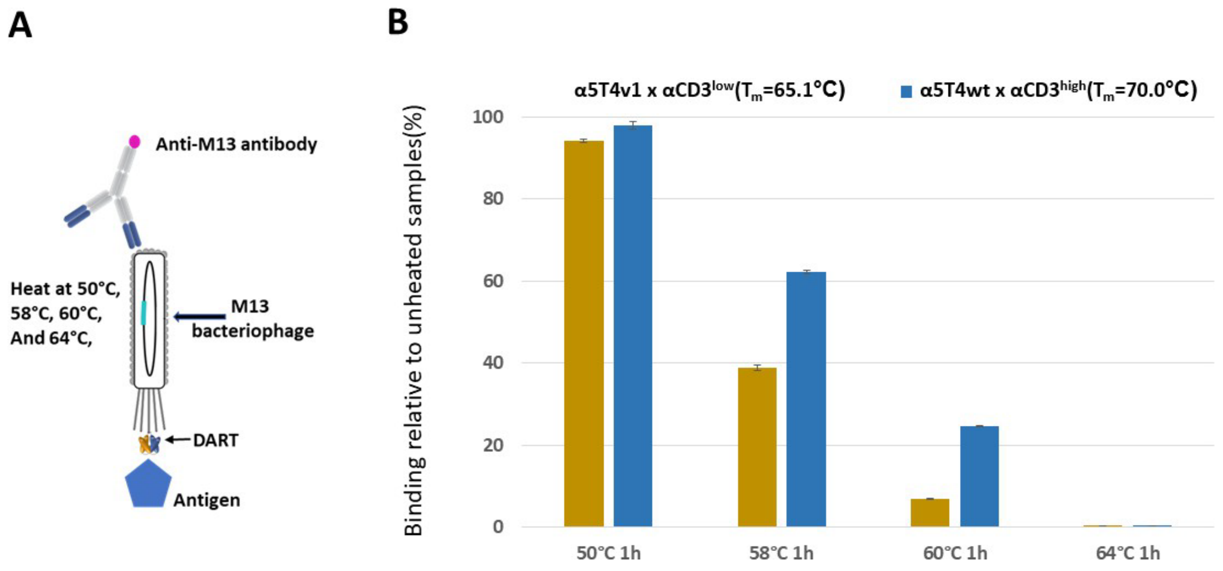

Figure S1. ELISA binding to human 5T4 protein with heated phage of two DART variants, α5T4wt x $\alpha CD3^{high}$ and α5T4v1 x $\alpha CD3^{low}$, to estimate their stability. **A.** A schematic of the phage ELISA. **B.** The histogram of the ELISA with heated phage particles. In the assays, as the heating temperature increased from 50 °C to 64 °C, the percentage of binding retained after heating is used to estimate the stability.

A

Binding of unheated phage to cyno-5T4

	1	2	3	4	5	6	7	8	9	10	11	12
A	1092	794	295276	697944	1118	864	638	245580	644	500	434	542
B	640664	1030	838	1226	864	588398	1050	1082	546	438	436	1378
C	1640	776	1156	682	638	964	946	625972	990	488	548	1786
D	939852	1698	977110	1412	604	546	664	920	820	772	540	1048
E	1364	850	1306	169436	1330	776	770	576	598	598	1466	1806
F	780	892	838	1132	966688	1380	680	1348	880	972	1118722	1004418
G	856	337054	904	1004	1468	728	1126	1097642	307158	942	1698	1452
H	900	1924	2616	322276	1022	712	832	1540	1064	916	301426	180368
									High-expression Ctr		α5T4v1 x αCD3^{low}	

Binding of heated (58°C, 1h) phage to cyno-5T4

	1	2	3	4	5	6	7	8	9	10	11	12
A	7640	4254	152944	172546	642	874	496	130228	574	424	1002	3160
B	331740	738	634	920	862	369814	740	822	504	462	540	478
C	1512	754	718	588	608	790	628	371682	810	474	452	446
D	625850	1322	414020	848	632	592	758	694	488	1830	536	562
E	1346	808	986	37176	1174	814	712	1828	858	882	1056	988
F	916	804	750	1382	852210	1320	660	1014	830	908	611880	481170
G	1598	91718	872	1202	1436	876	966	437622	150626	1078	1138	1752
H	3280	1230	916	238280	1078	878	1322	1226	1000	1888	130402	97172
									High-expression Ctr		α5T4v1 x αCD3^{low}	

Binding to an α-EK-coil antibody to measure DART expression

	1	2	3	4	5	6	7	8	9	10	11	12
A	870	698	5216	205560	708	590	640	119212	566	486	458	424
B	72526	658	574	854	592	98458	8866	592	450	444	532	430
C	950	776	1252	706	706	674	588	127274	706	568	540	544
D	145904	914	148300	980	714	920	748	820	636	866	762	672
E	1358	1162	53602	231886	1238	826	866	768	788	1742	896	758
F	3442	886	990	1268	244468	1050	714	952	1520	854	286076	165044
G	1398	77474	1398	1012	1116	828	1010	240390	132660	1052	1012	1410
H	1276	1088	1112	157628	1184	910	1064	1182	454418	815920	83942	57036
									High-expression Ctr		α5T4v1 x αCD3^{low}	

B

Binding of unheated phage to cyno-5T4 normalized to DART expression(α-EK-coil)

	1	2	3	4	5	6	7	8	9	10	11	12
A	1.3	1.1	56.6	3.4	1.6	1.5	1.0	2.1	1.1	1.0	0.9	1.3
B	8.8	1.6	1.5	1.4	1.5	6.0	0.1	1.8	1.2	1.0	0.8	3.2
C	1.7	1.0	0.9	1.0	0.9	1.4	1.6	4.9	1.4	0.9	1.0	3.3
D	6.4	1.9	6.6	1.4	0.8	0.6	0.9	1.1	1.3	0.9	0.7	1.6
E	1.0	0.7	0.0	0.7	1.1	0.9	0.9	0.8	0.8	0.3	1.6	2.4
F	0.2	1.0	0.8	0.9	4.0	1.3	1.0	1.4	0.6	1.1	3.9	6.1
G	0.6	4.4	0.6	1.0	1.3	0.9	1.1	4.6	2.3	0.9	1.7	1.0
H	0.7	1.8	2.4	2.0	0.9	0.8	0.8	1.3	0.0	0.0	3.6	3.2
									High-expression Ctrl		α5T4v1 x αCD3^{low}	

α5T4v3 x αCD3^{low} (Lead molecule)

An unrelated DART molecule of high expression (CHO cells, 918 mg/L) as a positive control of high phage expression

α5T4v1 x αCD3^{low} (Parent)

Figure S2. The ELISA chemiluminescent values and calculated ratios of ELISA assays, from which the lead molecule was selected. **A.** The ELISA chemiluminescent binding values of 92 variants selected from the 3rd round biopanning of library 1. Unheated phage supernatants were screened for binding to cyno 5T4 (top assay) and anti-E/K coil antibody (bottom assay; for quantifying DART expression on phage). Heated phage supernatants (58 °C for 1 hour) were screened for binding to cyno 5T4 (middle assay). The variant highlighted in magenta is the lead molecule, α5T4v3 x αCD3^{low}. Two phage controls were used in each assay: (i) phage displaying an irrelevant DART molecule with high expression, highlighted in blue; (ii) phage displaying parent α5T4v1 x αCD3^{low} as 5T4 binding control, highlighted in brown. **B.** Ratio of cyno 5T4 and anti-E/K-coil binding signals of unheated supernatants of selected variants.

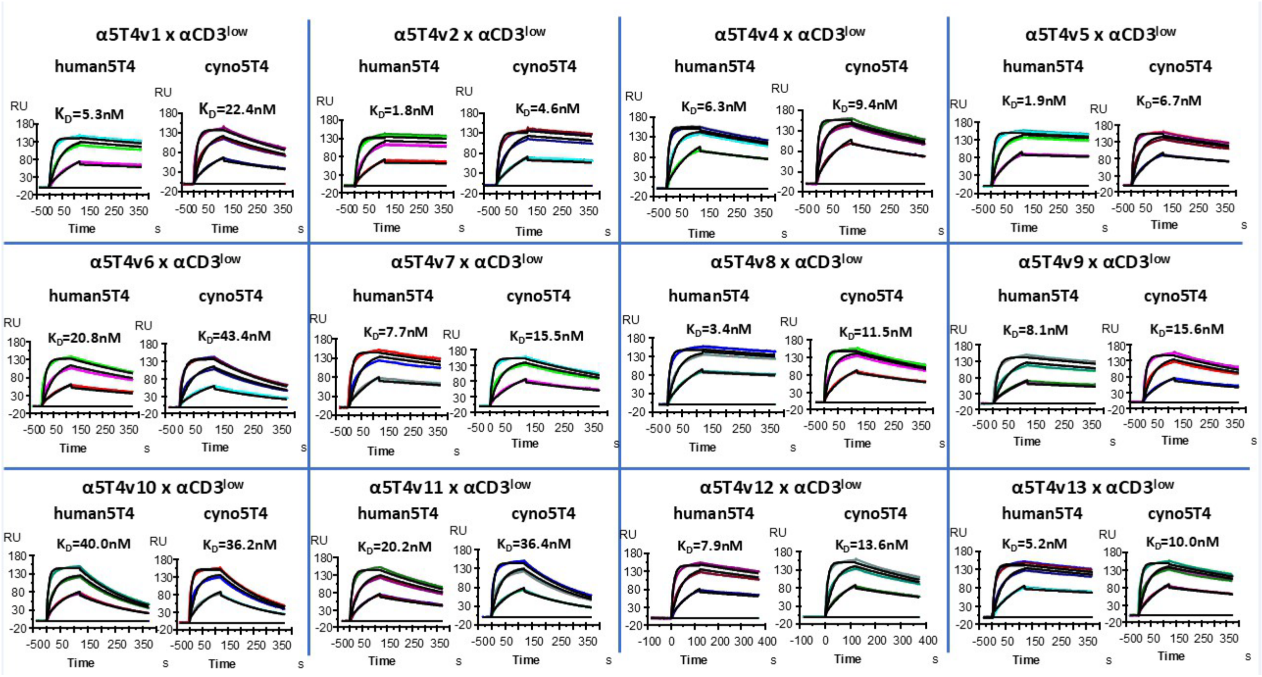

Figure S3. SPR sensorgrams for measuring the affinity of selected variants against human and cyno 5T4. A F(ab')2 fragment of goat anti-human IgG Fc antibody was immobilized on a CM5 sensor chip, followed by injection of DART molecules, and then human or cyno 5T4 protein at concentrations of 0, 62.5, 250, and

1000 nM. The dissociation equilibrium constant (K_D) was calculated by globally fitting the association/dissociation curves using the 1:1 Langmuir interaction model.

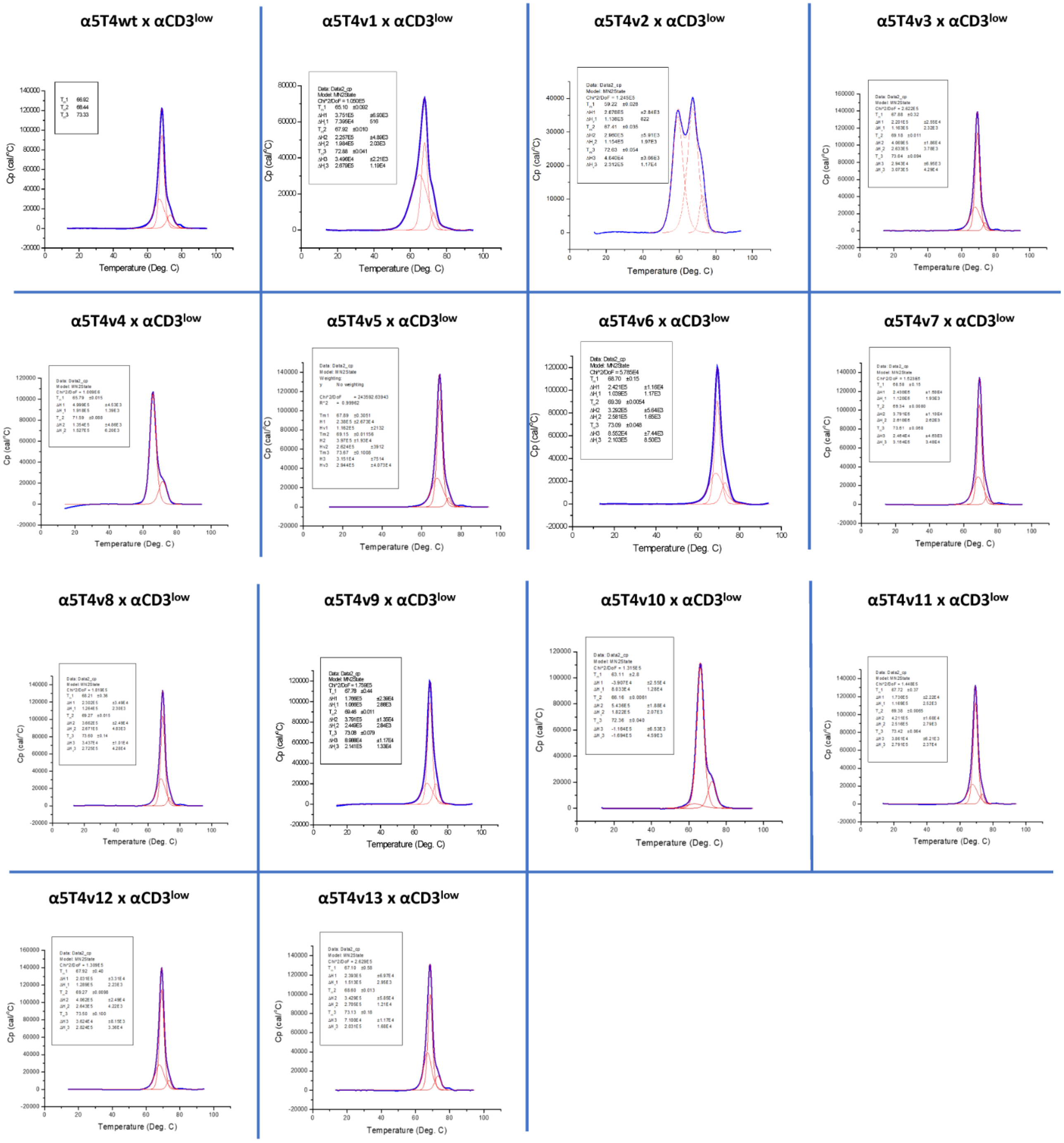

Figure S4. The thermograms for measuring the melting temperature (T_m) of selected variants by differential scanning calorimetry. Thermal scans of proteins at 1 mg/mL in PBS buffer were performed from 15 °C to 95 °C at a heating rate of 1 °C/min. Thermograms were obtained by subtracting the buffer scan and fitting to a non-2-state model.

A

VL variants	VH variants	0.5-mL culture expression (μg/mL)	560-mL culture expression (mg/L)	560-mL expression improvement over α5T4v2 (fold)	T_m (°C)
α5T4wt (VL-F36)	α5T4v1	16 ± 1.0	134	N/A	65.1
	α5T4v2	3.4 ± 0.4	55	N/A	59.6
	α5T4v3	62.5 ± 6.2	297	5.4	67.9
	α5T4v4	36.2 ± 0.9	440	8.0	65.8
	α5T4v5	40.9 ± 1.5	300	5.5	67.9
	α5T4v7	36.7 ± 0.6	243	4.4	68.6
	α5T4v8	33.5 ± 3.0	276	5.0	68.2
α5T4v14 (VL-F36L)	α5T4v1	17.6 ± 0.9	153	2.8	66.9
	α5T4v2	5.9 ± 0.3	78	1.4	61.9
	α5T4v3	41.4 ± 2.7	402	7.3	66.4
	α5T4v4	47.7 ± 2.3	333	6.1	67.4
	α5T4v5	57.5 ± 2.2	371	6.7	67.4
	α5T4v7	38.9 ± 0.9	260	4.7	67.3
	α5T4v8	34.5 ± 0.6	285	5.2	67.9

B

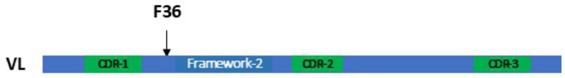

C

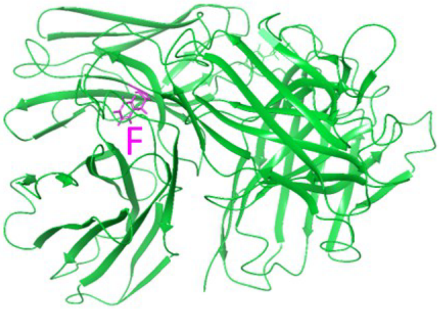

Figure S5. Improvement of expression levels by pairing different VH and VL variants and the computational model of the α5T4v2 x αCD3^{low} variant. **A.** A summary table listing expression levels from 0.5 mL and 560 mL cultures, expression fold improvement of variants over α5T4v2 x αCD3^{low}, melting temperatures (T_m) of α5T4 x αCD3^{low} DART variants. The 5 variants (v3-v8) selected from directed-mutation library (library 1) differed from the two parent molecules (v1 and v2) only in their heavy chain and share the same wild-type light chain (VL-F36). These 7 VH variants were combined with a new VL variant (VL-F36L) identified from the library of random mutations (Library 2) to create seven new DART molecules. The expression level of 0.5 mL culture was estimated by ELISA, whereas the expression of 560 mL culture was the amount of protein captured by protein-A column. The melting temperature (T_m) was measured by differential scanning calorimetry. **B.** Schematic showing the location of the single point mutation in the VL region of this VL-F36L variant. **C.** A computational homology model of α5T4v2 x αCD3^{low}, built by Schrodinger BioLuminate with the crystal structure of the PDB: 5FCS, a basic DART molecule of αP-cadherin x αCD3^{high}. Phenylalanine 36 (VL-F36) was highlighted in magenta.

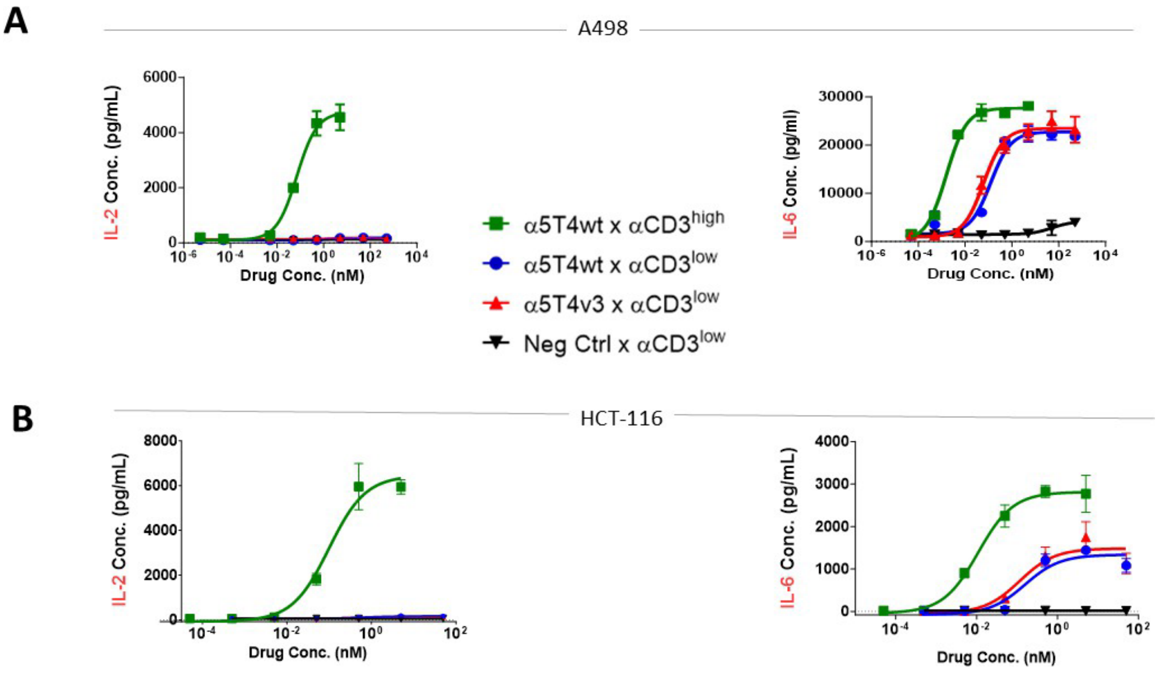

Figure S6. The release of cytokines, interleukin-2 (IL-2) and interleukin-6 (IL-6), induced by the DART-redirected T-cell killing of tumor cells. **A.** IL-2 and IL-6 secretion during T-cell mediated killing of A498 tumor cells. Four DART molecules were tested, including α5T4wt x αCD3^{high}, α5T4wt x αCD3^{low}, α5T4v3 x αCD3^{low} and a control DART molecule, αHIV-Env-gp120 x αCD3^{low}. Human peripheral blood mononuclear cells (PBMCs) were mixed with the tumor cells at the ratio of 10:1 and the assays lasted 48 hours prior to analysis. **B.** IL-2 and IL-6 release during T-cell mediated killing of HCT-116 tumor cells.

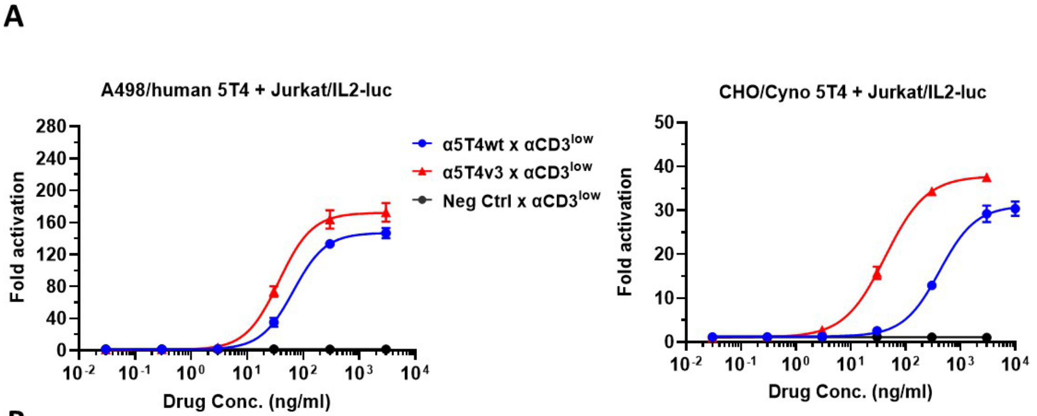

B

DART molecule	EC_{50} [ng/mL]	
	A498/ Human5T4	CHO/Cyno5T4
α5T4wt x αCD3^{low}	66.6	415.7
α5T4v3 x αCD3^{low}	34.0	42.6

Figure S7. CD3 signaling induced by 5T4 x CD3 DART molecules in a T cell activation reporter assay. A. Jurkat T cells with a luciferase reporter gene driven by an IL-2 promoter were incubated with A498 cells

expressing human 5T4 or CHO cells expressing cyno 5T4 in the presence of DART molecules. Data shown are the average of two experiments. B. The EC_{50} values of DART molecules in the reporter assay.

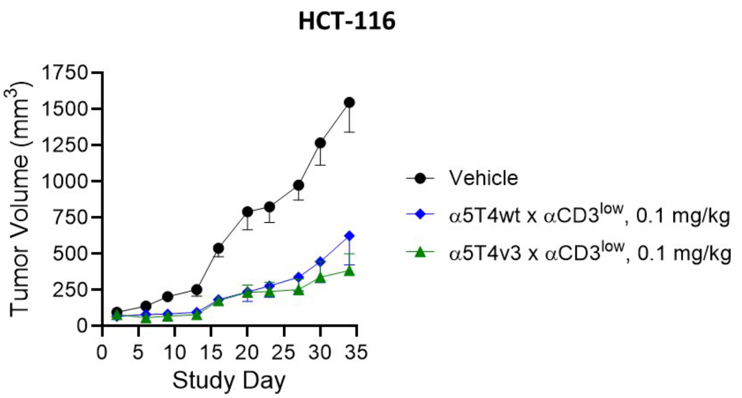

Figure S8. Anti-tumor activity of α5T4wt x $\alpha CD3^{low}$ and α5T4v3 x $\alpha CD3^{low}$ in HCT-116 cell line-derived xenograft models. Human PBMCs were injected into NSG/MHCI-/- mice at day 0, followed by injection of tumor cells at day 7. The DART molecules were administered at 0.1 mg/kg once a week starting at day 7.

Table S1. 5T4 expression on a panel of tumor cell lines and the cytotoxicity profile of α5T4v3 x $\alpha CD3^{low}$ in killing such tumor cells. The 5T4 expression across multiple tumor cell lines was measured by quantitative flow cytometry, which was carried out with the IgG form of α5T4wt conjugated to AlexaFluor488 (Concentration:10 µg/mL). Potency (EC_{50}) and maximum tumor cell lysis (E_{max}) of α5T4v3 x $\alpha CD3^{low}$ in redirected T-cell mediated killing of a panel of cancer cell lines. E_{max} and EC_{50} values are average values of at least 3 experiments.

Cell line	5T4 binding sites	EMax (%)	EC50 (nM)
A498	173811	36.6	0.03
MDA-MB-231	238432	27.7	0.02
786-0	286230	24.1	0.01
H1975	223349	24.0	0.05
HCT-116	152741	13.0	0.06
FaDu	213686	17.9	0.02
BxPC3	206459	15.8	0.03
H292	129559	13.5	0.02
A549	156215	11.5	0.05

Supplemental Methods and Materials

Computational modeling

Using the crystal structure of an αP-cadherin x $\alpha CD3^{high}$ DART molecule (PDB:5FCS) as a template, Schrodinger Bioluminate 4.6 (Schrodinger Inc) was used to generate a homology model of α5T4v2 x $\alpha CD3^{low}$. The sequences of two α5T4v2 x $\alpha CD3^{low}$ polypeptide chains were used as the input for generating the homology model. Once both chains of α5T4v2 x $\alpha CD3^{low}$ and their respective template were selected, the heterodimer modeling function was utilized to generate the model.

T-cell activation assays

T-cell activation assays were conducted using Jurkat T-cell lines expressing a luciferase reporter driven by an IL-2 promoter (Promega, cat#: J1651). In a 96-well plate, Jurkat T-cell lines (1×10^5/well) were cocultured with A498 cells or CHO cells expressing cyno 5T4 at 10:1 ratio in the presence of 10-fold serial dilution of test article. After 24 hours, T-cell activation was measured by reading the bioluminescence signal of the culture by a luminescence plate reader. The results shown were the average of two experiments.

Quantification of 5T4 antibody binding sites on tumor cell lines

A total of 1×10^6 cells of each tumor cell line were suspended in the flow cytometry buffer (PBS + 1% BSA + 0.1% sodium azide; BD Bioscience, cat#: 554657). Cells were then labeled with anti-5T4-Alexa 488 (clone h5E6, 10 µg/mL, MacroGenics) or isotype control antibody for 30 minutes at 4°C. After washing with flow cytometry buffer, labeled cells were resuspended in 0.4 mL buffer for flow cytometry acquisition. Microspheres with different antibody binding capacities (Quantum™ Simply Cellular®, QSC, Bangs Laboratories, cat#: 816) that were labeled with anti-5T4-Alexa488 antibody were used to generate a standard curve and calculate the number of 5T4 receptors on the cell lines from the fluorescent intensities.
